# Tetragonal distortion of the multiferroic compound α-LiFe_5_O_8_ from first-principles calculations

**DOI:** 10.1107/S1600576726006709

**Published:** 2026-07-31

**Authors:** Yaoshi Cheng, So-Hyun Park

**Affiliations:** a Applied Crystallography and Geomaterials, Department of Earth and Environmental Sciences Ludwig-Maximilians-Universität München Theresienstrasse 41C Munich Bavaria80333 Germany; Oak Ridge National Laboratory, USA

**Keywords:** α-LiFe_5_O_8_, ferrimagnetic multiferroics, band structure, first-principles calculations, density of states, Hubbard model, density functional theory, magnetic exchange interactions

## Abstract

The cubic space group *P*4_3_32 has been previously reported for the multiferroic lithium ferrite (α-LiFe_5_O_8_, denoted LFO). Instead, results from the present study using PBEsol+*U* calculations with SSSP pseudopotentials suggest that the tetragonal space group *P*4_3_2_1_2 is true for the ground state of LFO. Spin-resolved projected density of states analysis shows strong 3*d* electron localization of Fe^3+^ in FeO_4_ tetrahedra, mostly contributing to the high Hubbard energy *U* in the calculated tetragonal model. Its direct band gap of 2.050 eV is slightly lower than that of the cubic model (2.107 eV) in density of states calculations. The calculated ferrimagnetic ground state exhibits a collinear spin order along the crystallographic *c* axis in the tetragonal magnetic space group *P*4_3_2_1_′2′, consistent with the group theoretical prediction. Decreasing ferromagnetic interactions and increasing antiferromagnetic interactions in the *c* direction, simultaneously, explain the origin of the tetragonality *a*/*c* > 1 with *a* = 8.3528 (2) Å and *c* = 8.3511 (1) Å. Strong Fe–O–Fe superexchange interactions contribute to the tetragonal lattice distortion of LFO as well.

## Abstract 2

First-principles calculations suggest the tetragonal space group *P*4_3_2_1_2 for the multiferroic compound α-LiFe_5_O_8_, with a direct band gap smaller than that in its widely known cubic space group *P*4_3_32. The tetragonal lattice distortion *a* > *c* is explained by relatively strong antiferromagnetic exchange interactions parallel to the *c* axis and by Fe–O–Fe magnetic superexchange interactions, consistent with Fe 3*d*–O 2*p* hybridization.

## 1. Introduction

The photovoltaic (PV) effect in magnetoelectric (ME) multiferroics (MFs) has been increasingly investigated by finding spintronics showing helical magnetic spin order-induced electric polarization (Young *et al.*, 2013 ▸; Xiao *et al.*, 2023 ▸; Xu *et al.*, 2021 ▸). The bulk PV effect in ME-MFs occurs with photon-excited electrons jumping from the valence band to the conduction band under inherent electric fields in their polar electronic structures due to the magnetic spin order-induced breaking of space inversion (Baltz & Kraut, 1981 ▸). Thus, in contrast to p-n-junction semiconductor PVs, the resulting photovoltage in ME-based PVs is barely associated with the band-gap energy (Rangel *et al.*, 2017 ▸; Jalaja & Dutta, 2015 ▸). In ME-MFs, the coexistence of ferroelectricity and magnetic spin order allows adjustment of their PV performance by applying external magnetic and electric fields. Furthermore, the MF-PV effect can be significantly enhanced by mechanical and chemical stress using thin films and doping (Vopson, 2015 ▸; Dharmadhikari & Grannemann, 1982 ▸; Ji *et al.*, 2010 ▸; Peng *et al.*, 2017 ▸; Lee & Son, 2024 ▸). Hence, MFs can be photoactive over a wide solar spectrum. In particular, thin films of ME-MFs such as BaTiO_3_ (Koch *et al.*, 1975 ▸), BiFeO_3_ (Yang *et al.*, 2009 ▸) and Bi_2_FeCrO_6_ (Mahapatra *et al.*, 2024 ▸) have been given attention for PV applications due to their strong response to photons. Their relatively low band gaps of about 2.04–3.21 eV are advantageous compared with those of pure ferroelectric PVs (Nechache *et al.*, 2015 ▸; Jalaja & Dutta, 2015 ▸). For the bulk PV effect, the photocurrent contributes to the shift current induced by electric polarization, as well as to the ballistic current induced by momentum asymmetry of photon-excited carriers (Burger *et al.*, 2019 ▸). Therefore, a low band-gap energy for MFs is desired for strong electron–electron interactions to overcome their photocurrent upper limit of milliamps per square centimetre in contrast to the limit of several thousand amps per square centimetre for semiconductor-based PVs (Jalaja & Dutta, 2015 ▸; Huang *et al.*, 2021 ▸; Nakamura *et al.*, 2024 ▸; Dhass *et al.*, 2020 ▸). MF-PVs with a low band-gap energy will be particularly effective for the infrared region from 1.24 meV to 1.7 eV, which is the major part of the solar energy spectrum (Jalaja & Dutta, 2015 ▸; Nechache *et al.*, 2015 ▸).

Among MFs, the ordered phase of lithium ferrite [α-LiFe_5_O_8_ (Liu *et al.*, 2019 ▸); LFO hereafter] is interesting for its low band-gap energy depending on preparation: 1.67 and 1.86 eV for nest-like crystallites with and without using a reduced graphene oxide substrate, respectively (Zhang & Zhang, 2016 ▸), 2.37 eV for a nanocrystalline film on a glass substrate (Babrekar & Jadhav, 2017 ▸), and 1.40–1.60 eV for mixed nanoparticles of α-LiFe_5_O_8_ and β-LiFe_5_O_8_ calcined between 973 and 673 K (Chireh & Naseri, 2019 ▸). On the other hand, it is desirable to introduce a high ME coupling effect into multiferroics, preferably at room temperature for PV applications. In this sense, LFO is interesting for its high ME coupling coefficient (α_ME_) value of 2 mV Oe^−1^ cm^−1^ at 120 K; its α_ME_ value decreases with increasing temperature (0.2 mV Oe^−1^ cm^−1^ at 250 K), but the ME effect in LFO does exist up to room temperature (Liu *et al.*, 2019 ▸). The ferrimagnetic state of LFO is stable below its high Curie temperature of 907 K (Astafyev *et al.*, 2019 ▸), exhibiting a saturation magnetization of 2.5 μ_B_ per formula unit at room temperature (Pachauri *et al.*, 2015 ▸). This significant effective ME effect in LFO is attributed to the spin–orbital (SO) coupling of Fe^3+^ (3*d*^5^) residing in geometrically distorted octahedra (Mohapatra & Dobbidi, 2023 ▸). The resulting polyhedral distortion-induced electric polarization in a weak ferromagnetism (Mercier *et al.*, 1977 ▸) can be tuned by various external fields (Liu *et al.*, 2019 ▸).

LFO has been intensively investigated in both research and development of functional materials (Iliev *et al.*, 2011 ▸; De Picciotto & Thackeray, 1986 ▸; Gridnev *et al.*, 1997 ▸; Kinoshita *et al.*, 2016 ▸; Teixeira *et al.*, 2021 ▸; Soreto *et al.*, 2017 ▸; Kumawat *et al.*, 2021 ▸; Kim *et al.*, 2022 ▸; Sousa *et al.*, 2019 ▸; Moore *et al.*, 2024 ▸; Tan *et al.*, 2021 ▸). All these experimental and theoretical studies of LFO have been based on the atomic arrangements in the cubic space group *P*4_3_32 (Liu *et al.*, 2019 ▸). Its point group 432 is not a polar group and hence it disallows spontaneous electric polarization but allows the second-order ME effect. There are several unclear issues about its cubic space-group symmetry *P*4_3_32 and the consequent magnetic spin order in the cubic lattice (Kumawat *et al.*, 2021 ▸; Liu *et al.*, 2019 ▸). In particular, several strong magnetic Bragg reflections forbidden to its ferrimagnetic order in the cubic magnetic space group *P*4_3_32 were excluded in Rietveld refinements using high-resolution neutron powder diffraction (HRNPD) data (Kumawat *et al.*, 2021 ▸). By coincidence, the resulting collinear ferrimagnetic spin order is consistent with a theoretical magnetic spin order along the *c* axis predicted from *ab initio* calculations, where only the cubic space group *P*4_3_32 was considered (Kim *et al.*, 2022 ▸). However, according to the group–subgroup relation between space group and magnetic space group in group theory, the collinear spin arrangement is best realized in the tetragonal magnetic space group *P*4_3_
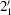
2′, the same as the ferrimagnetic structure of Al^3+^-doped LFO-type compounds in our recent HRNPD study (Inckemann *et al.*, 2025 ▸).

To date, there are no experimental band-gap energy data for LFO samples without microstructural effects. Results from previous density functional theory (DFT) calculations on LFO by different researchers are also contradictory to each other as they reported different band-gap energy values: Sousa *et al.* (2019 ▸) did not consider short-range interactions between localized Fe 3*d* electrons, giving rise to unreasonable valence band characteristics for LFO and an overestimated indirect band gap of 2.3 eV. Kim *et al.* (2022 ▸) calculated structural, electronic and magnetic properties of LFO in combination with the Hubbard model, resulting in a collinear ferrimagnetic spin configuration with a indirect band gap of 1.99 eV. However, in their DFT calculations, the Hubbard value *U* for the on-site Coulomb correction was determined merely by fitting the cubic lattice constant, rather than using the linear response method regarded as the standard way for obtaining *U* values (Moore *et al.*, 2024 ▸). For this reason, the underestimated interaction between Fe 3*d* electrons might be corrected further. According to the simulations done by Tan *et al.* (2021 ▸), the *U* value was set at 5.3 eV, where the valence band maximum was higher than the Fermi energy level. This could not be explained in terms of semiconductor behaviour. Thus, it is necessary to re-examine the true atomic and magnetic structures of LFO, which involves a symmetry lowering from cubic to tetragonal. The present study is an extensive re-investigation of the electronic structures of LFO in both cubic and tetragonal models using the DFT method with the Hubbard model. The simulated atomic and magnetic structures, as well as the electronic properties, agree with the tetragonal space group *P*4_3_2_1_2 being true for LFO, as described in the following.

## 2. Starting models

**Figure 1:**
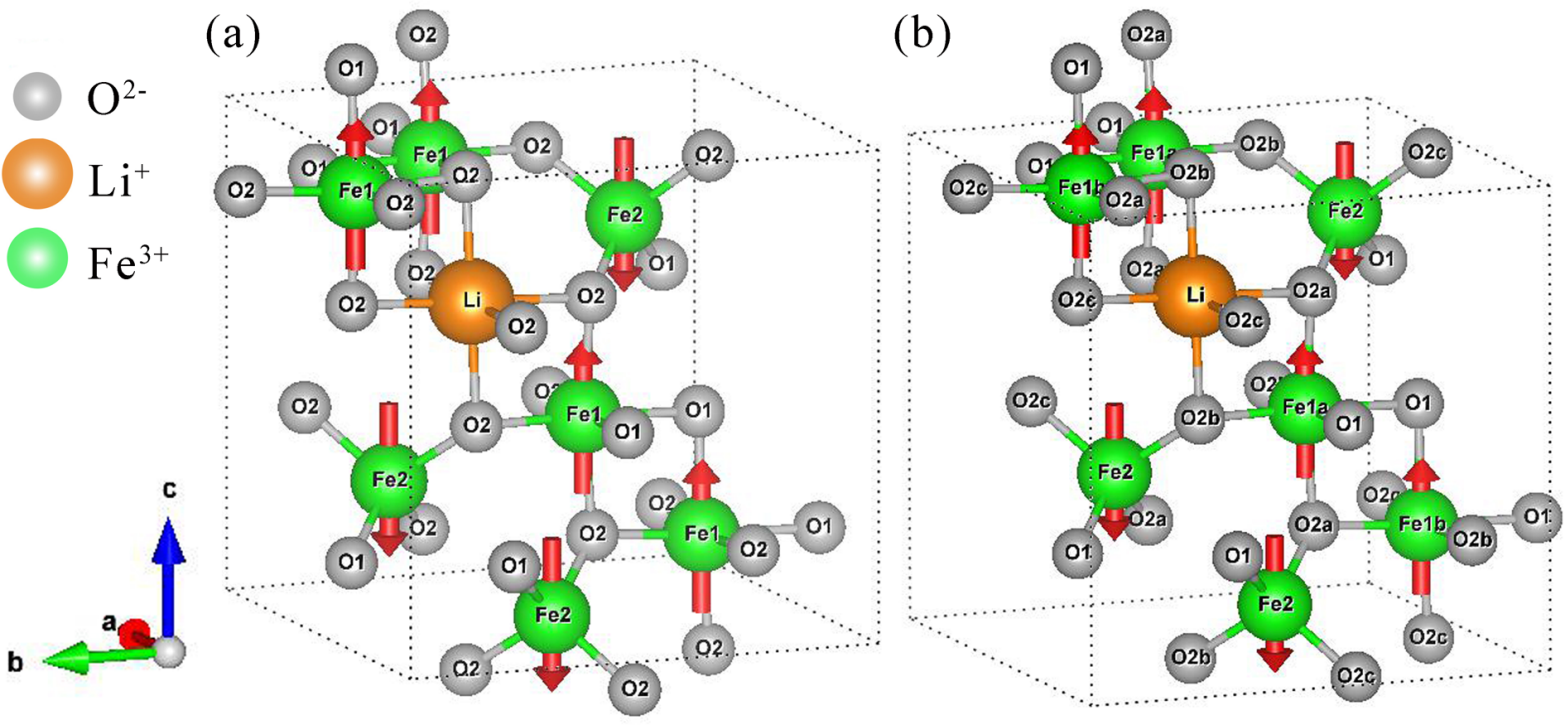
Initial models of α-LiFe_5_O_8_, (*a*) in *P*4_3_32 and (*b*) in *P*4_3_2_1_2 with site splitting for DFT simulation. For both starting models, a collinear ferrimagnetic spin order is configured with two sublattices of Fe^3+^ in FeO_4_ and FeO_6_ polyhedra.

Following previous experimental and theoretical studies of LFO (Kim *et al.*, 2022 ▸; Kumawat *et al.*, 2021 ▸), we have taken a collinear ferrimagnetic spin configuration of Fe^3+^ in the tetragonal and octahedral sublattices in two starting models:

(i) The well known cubic model in *P*4_3_32. Li^+^ is located on an octahedral site (4*b*) while Fe^3+^ is found on both octahedral (12*d*) and tetrahedral (8*c*) sites. The respective Fe sites are labelled Fe1 and Fe2 [Fig. 1 ▸(*a*)].

(ii) The tetragonal model in *P*4_3_2_1_2, a direct subgroup of *P*4_3_32. Its magnetic subgroup *P*4_3_
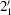
2′ is the only possible one to allow a collinear ferrimagnetic arrangement along *c* among the cubic (*P*
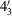
32′ and *P*4_3_32) and tetragonal (*P*4_3_
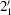
2′, *P*
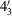

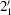
2, *P*
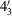
2_1_2′ and *P*4_3_2_1_2) magnetic subgroups of *P*4_3_32 [see the supporting information of Inckemann *et al.* (2025 ▸)]. This tetragonal distortion is accompanied by splitting on the Fe1 and O2 sites, *i.e.* the octahedral site for Fe (12*d*) and the site for O (24*e*) in the cubic model are split into Fe1*a* (8*b*) + Fe1*b* (4*a*) and O2*a* (8*b*) + O2*b* (8*b*) + O2*c* (8*b*), respectively, in the tetragonal model [Fig. 1 ▸(*b*)].

**Figure 2:**
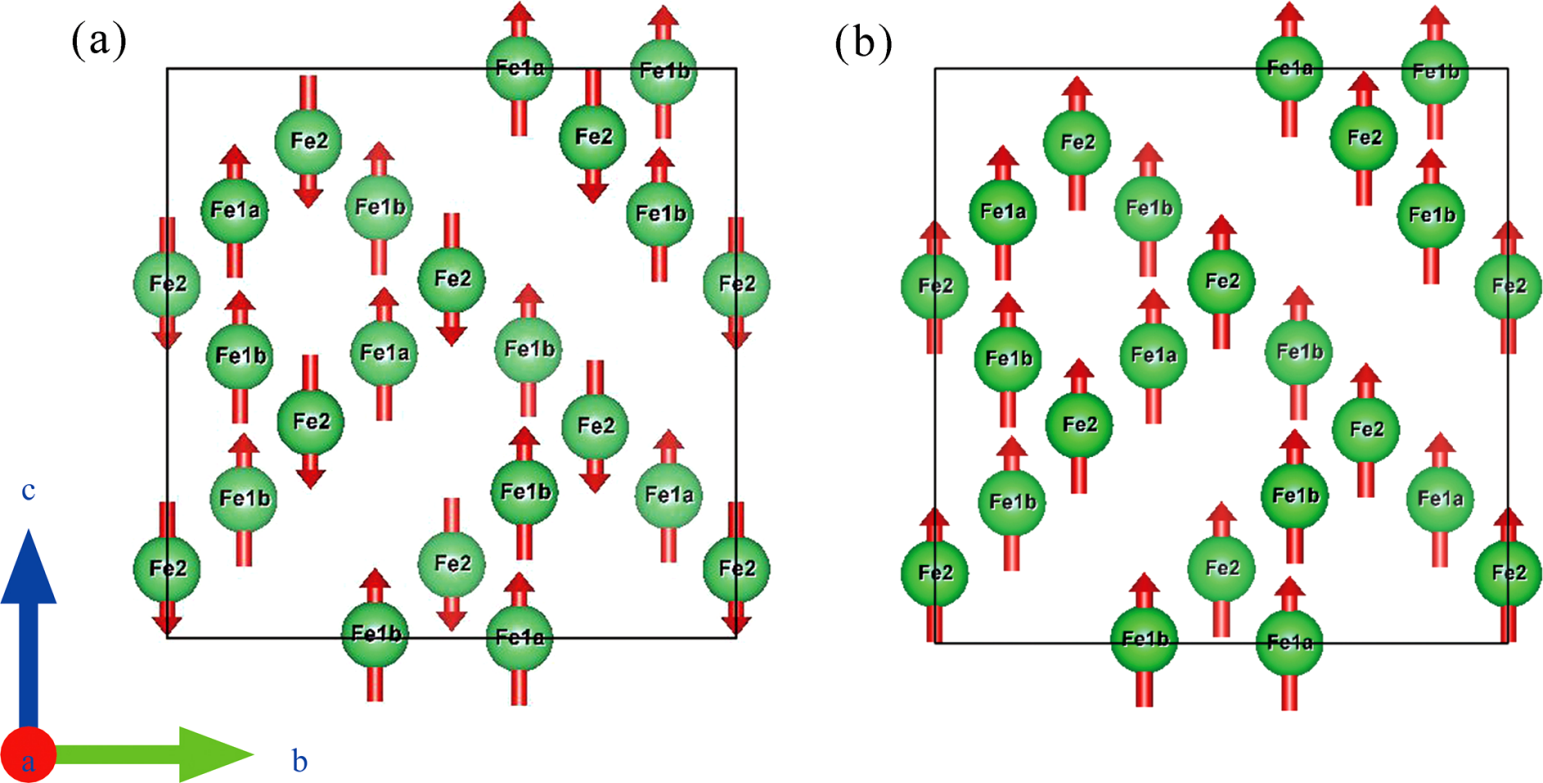
Two theoretical (*a*) ferrimagnetic and (*b*) ferromagnetic arrangements of Fe^3+^ in the starting models for the ground state of α-LiFe_5_O_8_ in *P*4_3_2_1_2.

Using all the atomic parameters including magnetic moments, *U* values were fitted for all Fe^3+^ ions for both models. To prove the difference between the ferri- [Fig. 2 ▸(*a*)] and ferromagnetic [Fig. 2 ▸(*b*)] ground states, we calculated their total energies.

## 3. Computational methods

DFT, based on the electron density and exchange-correlation approximations, explains the behaviour of the electrons and atomic nuclei of the investigated system by solving the Kohn–Sham equation without any experimental data (Bagayoko, 2014 ▸). As a reliable first-principles calculation method, DFT has contributed greatly to predicting and explaining coupling effects in various types of multiferroic materials beyond their structural and electronic properties (Neaton *et al.*, 2005 ▸; Ederer & Spaldin, 2006 ▸). Combined with the first-order perturbation theory, the density functional perturbation theory (DFPT) allows the simulation, with high computational efficiency, of crystal systems with a localized perturbation in one unit cell without requiring a supercell (Baroni *et al.*, 2001 ▸).

In the present study, for the multiferroic state of LFO, the magnetic spin of Fe^3+^ has been considered with the valence electronic configurations of Fe (3*p*^6^3*d*^5^4*s*^2^), Li (1*s*^2^2*s*^1^) and O (2*s*^2^2*p*^4^). All Fe^3+^ species in LFO exhibit the high-spin state (*S* = 5/2). Strong correlations between the Fe 3*d* electrons were described by the Hubbard model. The DFPT+*U* method was applied to correct the on-site Coulomb interaction *U* and spin polarization (Anisimov *et al.*, 1991 ▸), where *U* describes the potential energy of a 3*d*^5^ electron. In Hubbard-corrected DFPT calculations, the total energy of a spinel crystal system *E*_DFPT+*U*_ is given by the sum of the energy without spins (*E*_DFPT_) plus the energy of the on-site Coulomb correction term with a spin contribution (*E*_*U*_), 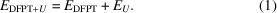
In order to determine *U*, DFPT calculations with the first-order linear-response theory in reciprocal space were self-consistently performed by the *HP* code (Timrov *et al.*, 2022 ▸). The convergence threshold for the response functions was set to 1 × 10^−6^ eV^−1^.

With the corrected *U* values, DFT calculations were executed using the *Quantum ESPRESSO* software suite (Giannozzi *et al.*, 2009 ▸; Giannozzi *et al.*, 2017 ▸) with the standard solid-state pseudopotentials (SSSP) (Version 1.3.0; Pran­dini *et al.*, 2018 ▸). Using the Perdew–Burke–Ernzerhof (PBE) (Perdew *et al.*, 1996 ▸) method for the generalized gra­dient approximation, exchange-correlation functionals were implemented to optimize the lattice metric and atomic parameters, and subsequently to calculate band structures. The lattice parameters are strongly related to magnetic prop­erties, such as magnetic exchange interactions. Hence, we applied the PBEsol method to DFT calculations to improve the accuracy of the lattice parameters (Perdew *et al.*, 2008 ▸). The kinetic energy cutoff for wavefunctions was tested and could be set to 100 Ry. According to the Monkhorst–Pack scheme, the *k*-point meshes in the Brillouin zone were sampled by 5 × 5 × 5 grids for both cubic and tetragonal unit cells of LFO. The total energy convergence criterion of 1 × 10^−6^ Ry and the forces convergence threshold of 1 × 10^−4^ Ry per Bohr were parameterized. The charge density cutoff of 1100 Ry was set when using SSSP to describe details in orbital electronic structures near the iron nucleus (Prandini *et al.*, 2018 ▸).

To explain the origin of tetragonality for LFO, magnetic exchange interactions were calculated with the Green function method according to Liechtenstein–Katsnelson–Antropov–Gubanov (LKAG) (Liechtenstein *et al.*, 1987 ▸). For a classical Heisenberg model, the Hamiltonian is described as
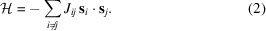
*J*_*ij*_ is an exchange interaction between two magnetic moments on sites *i* and *j*; and **s**_*i*_ and **s**_*j*_ are the unit vectors pointing in the magnetic moment direction on sites *i* and *j*, respectively. The magnetic exchange parameters were calculated on the basis of the magnetic force theorem, while the change in electronic single-particle energies of the system was related to the perturbation in the magnetic spin subsystem. Accordingly, the exchange parameters *J*_*ij*_ could be described by the intersite Green functions. In DFT calculations, plane-wave-based methods were used to obtain electronic band structures with Bloch states. The resulting Bloch states wavefunctions were projected onto a localized basis (Wannier functions) using the *Wannier90* code (Pizzi *et al.*, 2020 ▸) for calculating *J*_*ij*_, where Wannier functions construct the effective Hamiltonian and give the intersite Green functions. Finally, the magnetic exchange parameters in real space were obtained from DFT calculations using the *TB2J* package (He *et al.*, 2021 ▸).

## 4. Results and discussion

### 4.1. Assessing the ground-state structures and on-site Hubbard parameters

**Figure 3:**
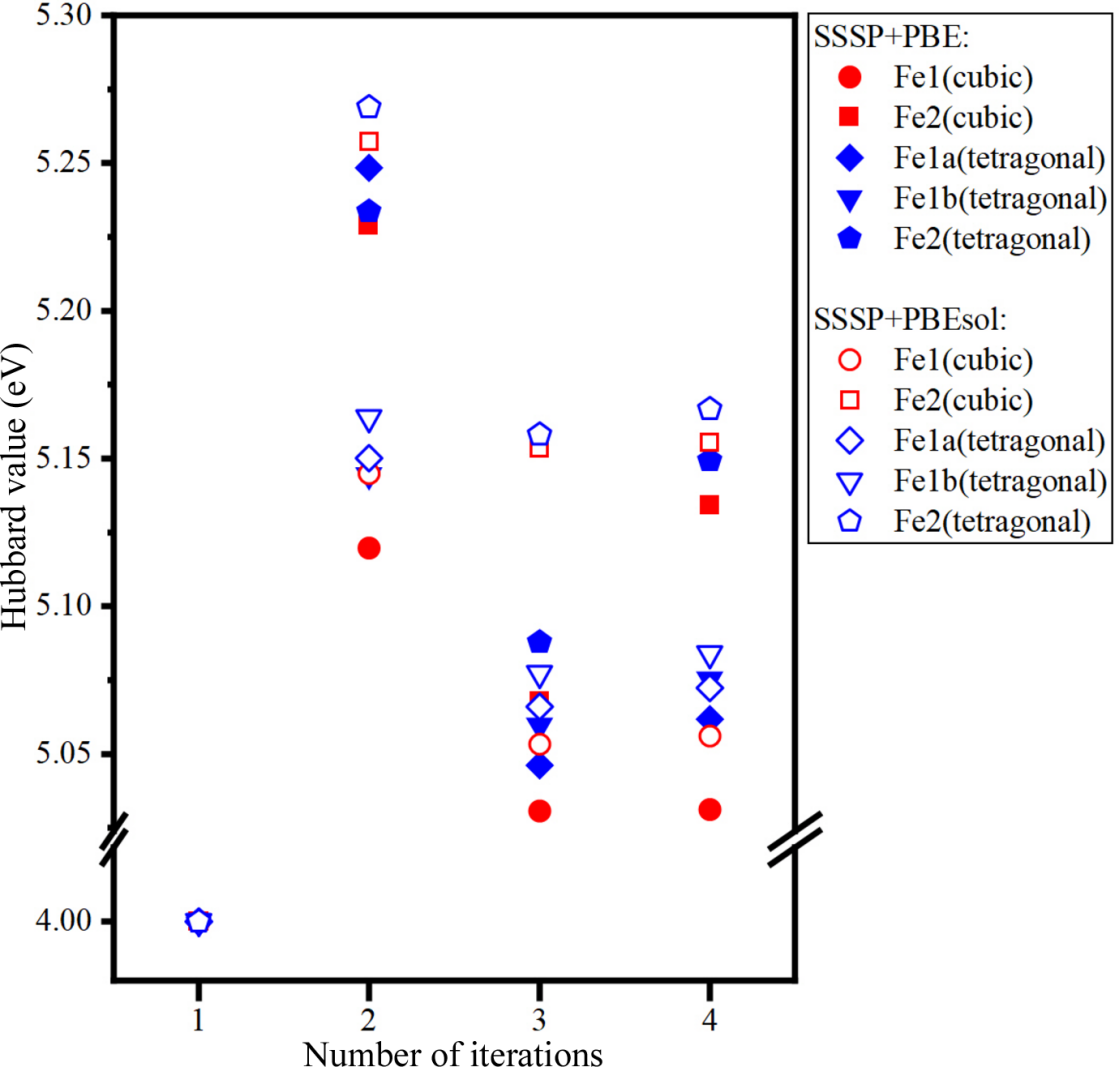
Hubbard values (*U*) calculated in this study for the cubic and tetragonal models of LFO in each iteration step.

The Hubbard *U* values were calculated on the tetrahedrally and octahedrally coordinated sites for Fe^3+^ in *P*4_3_32 and *P*4_3_2_1_2. The calculated *U* values between 5.0308 and 5.1668 eV from both SSSP+PBE (Table A.I in the supporting information) and SSSP+PBEsol (Table 1 ▸) methods agree with those typical for Fe^3+^ in various iron compounds (Moore *et al.*, 2024 ▸). Overall, as demonstrated in Fig. 3 ▸, the *U* values from the two methods are close to each other after the fourth iteration. The *U* values of Fe^3+^ in the tetragonal lattice in *P*4_3_2_1_2 are larger than those in the cubic lattice in *P*4_3_32, and this is especially significant on the tetrahedral site Fe2. Furthermore, this on-site Coulomb repulsion on Fe2 (*U* = 5.1668 eV) is stronger than those on the octahedral sites Fe1*a* (*U* = 5.0724 eV) and Fe1*b* (*U* = 5.0839 eV). This implies more localized Fe 3*d* electrons in FeO_4_ tetrahedra, particularly in the tetragonally distorted lattice (Table 1 ▸).

**Figure 4:**
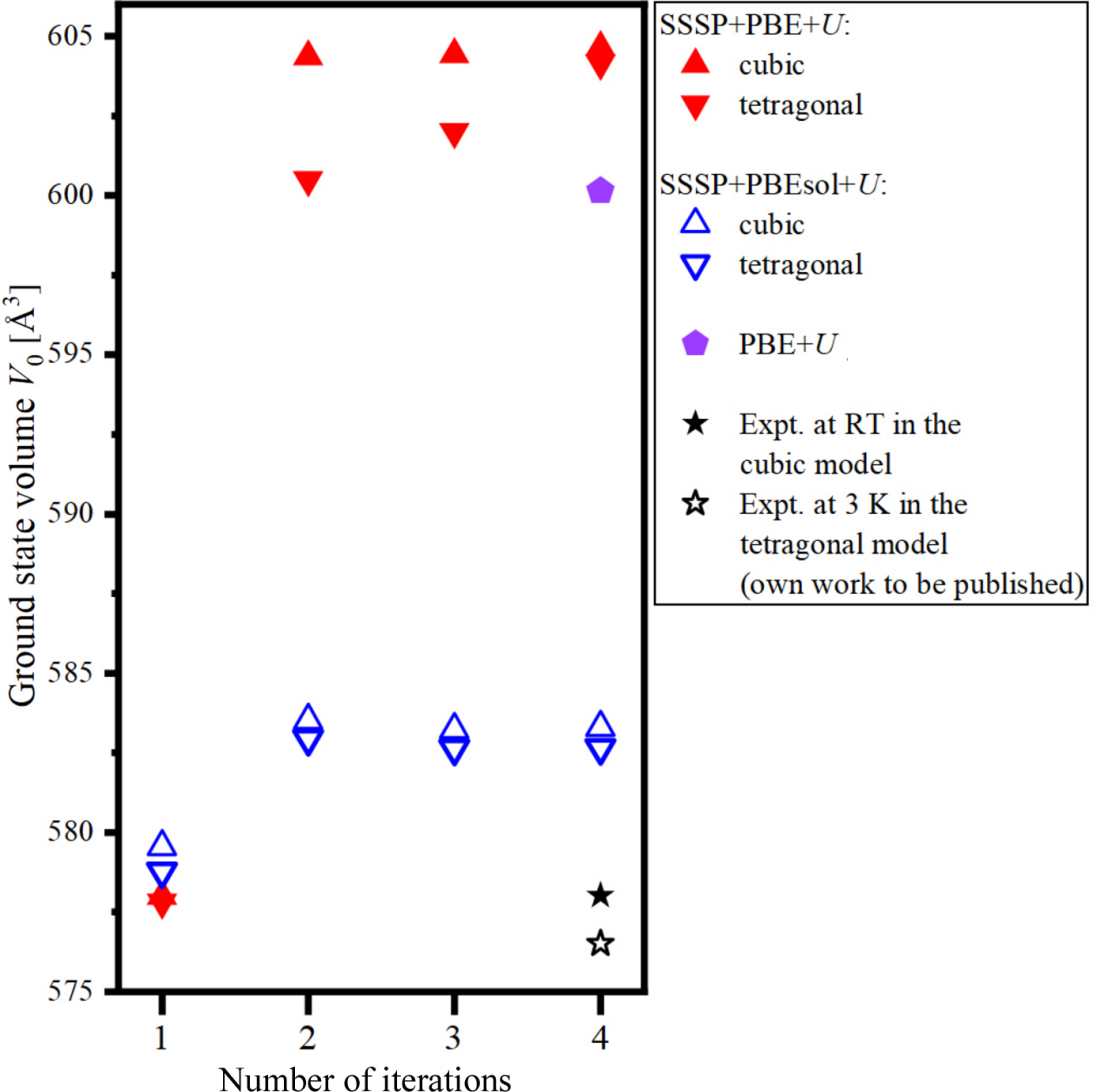
*V*_0_ values (triangles) calculated in this study for the cubic and tetragonal models of LFO in each iteration step. The volumes of LFO in the cubic space group reported by other experimental [filled star: Liu *et al.* (2019 ▸); open star: our work] and theoretical [pentagon: Tan *et al.* (2021 ▸)] studies are given for comparison.

From DFT calculations with the *U* values from both SSSP+PBE (Table A.I in the supporting information) and SSSP+PBEsol (Table 1 ▸) methods, the magnitudes of the total energy *E* for the cubic and tetragonal models were computed as a function of the unit-cell volume *V*, as shown in Fig. 4 ▸. The ground-state unit-cell volume (*V*_0_) and its total energy (*E*_0_) were estimated by least-squares fitting of the *E*–*V* curve in the third-order Birch–Murnaghan equation of state (EOS) (Birch, 1947 ▸),
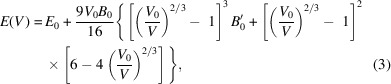
where *B*_0_ is the ground-state bulk modulus and 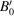 is the derivative of the bulk modulus against pressure. After four iteration steps of the geometry optimization, the value for *V*_0_ tends toward convergence, particularly by applying SSSP pseudopotentials with PBEsol exchange-correlation functionals (Fig. 4 ▸). The obtained *V*_0_ values of 583.2293 (3) and 582.6501 (3) Å^3^ for the cubic and tetragonal models, respectively, are comparable to experimental results, *e.g.V*_exp_ = 578.01 Å^3^ at room temperature for the cubic model (Liu *et al.*, 2019 ▸), while for the tetragonal model (our own work, to be published elsewhere), we obtained *V*_exp_ = 578.19 (2) Å^3^ at room temperature using X-ray single-crystal diffraction and *V*_exp_ = 576.49 (1) Å^3^ at 3 K by Rietveld analysis with HRNPD data. There is an extremely small difference of 0.1% in the *V*_0_ values between the tetragonal and cubic models, where the former with *a* > *c* is relatively close to *V*_exp_ values. In comparison, the *V*_0_ values from the SSSP+PBE method are much further from these experimental observations, as clearly seen in Fig. 4 ▸. The current study shows that the PBEsol exchange-correlation functional is suitable for calculating the ground-state unit-cell volume *V*_0_. Thus, we consider exclusively the results from the SSSP+PBEsol method hereafter. For the same reason, we employed the *U* values obtained using the SSSP+PBEsol method in the subsequent calculations of electronic band structures and magnetic interaction parameters (Sections 4.2 and 4.3).

The atomic positions were relaxed by geometry optimization iterations using the PBEsol method. The optimized structure parameters, bulk moduli and total energies are listed in Table 1 ▸. The tetragonal model of LFO shows an insignificantly lower total energy than the cubic model, *i.e.* a difference of 5 × 10^−4^ Ry, but with a high accuracy of 10^−9^ Ry. On the other hand, our calculations of the respective total energies −7911.0057 and −7910.4706 Ry for the respective ferri- and ferromagnetic orders in the tetragonal lattice (Fig. 2 ▸) point to the ferrimagnetic ground state in the tetragonal structure of LFO. As mentioned above, this ground-state ferrimagnetic spin order cannot be realized in the cubic magnetic subgroups of *P*4_3_32; the collinear ferrimagnetic spin arrangements along the crystallographic *c* axis are allowed exclusively in the tetragonal magnetic space group *P*4_3_
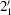
2′ (Inckemann *et al.*, 2025 ▸). Hence, a reasonable conclusion can be drawn that LFO undergoes a slight tetragonal lattice distortion. This is highly possible due to the magnetic origin, *i.e.* the small tetragonality [*a* = 8.3528 (2) Å, *c* = 8.3511 (1) Å] may arise from weak antiferromagnetic interactions between the tetragonal and octahedral sublattices of Fe^3+^, which compete with the ferromagnetic interactions within each sublattice (Fig. 2 ▸). This issue is addressed quantitatively in Section 4.3. The bulk modulus of 166.20 GPa calculated by the PBEsol method for the tetragonal model is consistent with the experimental value reported by Massoudi *et al.* (2020 ▸) as well. Overall, the current study suggests the ground-state structure of LFO is tetragonal in *P*4_3_2_1_2. This finding was applied in the subsequent calculations of band structures in Section 4.2 and magnetic interactions in Section 4.3.

**Figure 5:**
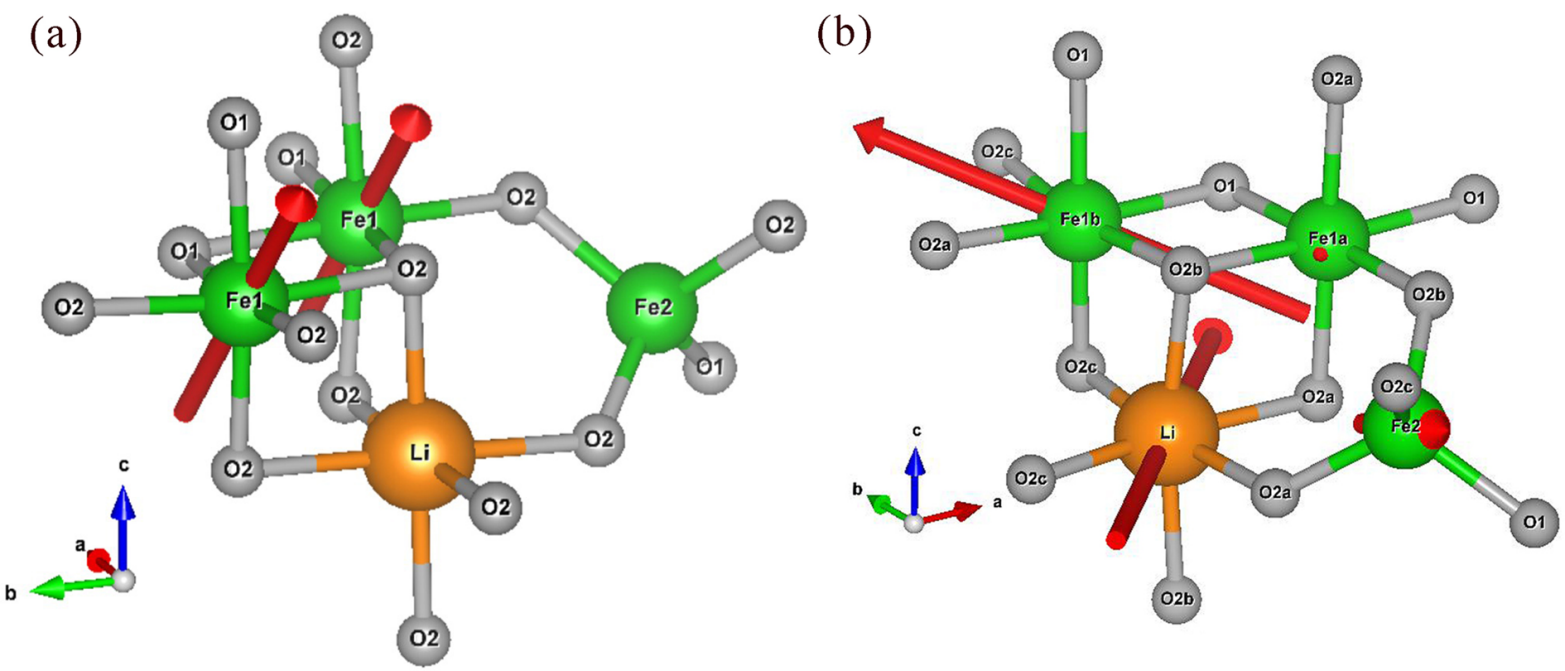
Polyhedral distortion-induced electric dipole moments (Table 2 ▸) are indicated by bold red arrows, (*a*) in the cubic model in *P*4_3_32 and (*b*) in the tetragonal model in *P*4_3_2_1_2. Both models were simulated by the PBEsol method.

Comparing the local structures of LiO_6_, FeO_6_ and FeO_4_ polyhedra (Fig. 5 ▸), their volumes, geometric degrees of distortion and distortion-induced electric dipole moments were evaluated using the best-fitted idealized polyhedron method (Song & Liu, 2019 ▸; Song *et al.*, 2023 ▸) (Table 2 ▸). Their degrees of polyhedral distortion and distortion-induced electric dipole moments (Fig. 5 ▸) show noticeable differences between the two models within calculation uncertainties (Table 2 ▸). In the tetragonal model, the small volume of FeO_4_ with obviously short Fe—O bonding distances (Tables A.I and A.II) reflects strong interactions between Fe on Fe2 with four ligand oxygens. The electric dipole moment modulus of FeO_4_ is negligibly small compared with those of highly distorted FeO_6_, particularly on Fe1*b*. The shrinkage in FeO_4_ tetrahedra in the tetragonal model is consistent with the higher *U* value on Fe2 compared with that in the cubic lattice. This hints at the presence of competing interactions, such as hybridization between O 2*p* and Fe 3*d* orbitals in a rigid and small FeO_4_ unit in the tetragonal ground state of LFO (see Section 4.2). The significantly different degrees of distortion between Fe1*a*O_6_ and Fe1*b*O_6_ [Fig. 5 ▸(*b*)] are a strong sign for lattice distortion towards a tetragonal lattice. Otherwise, in the cubic structure model, there may not be any differences between these two octahedra.

### 4.2. Electronic band structures and density of states

**Figure 6:**
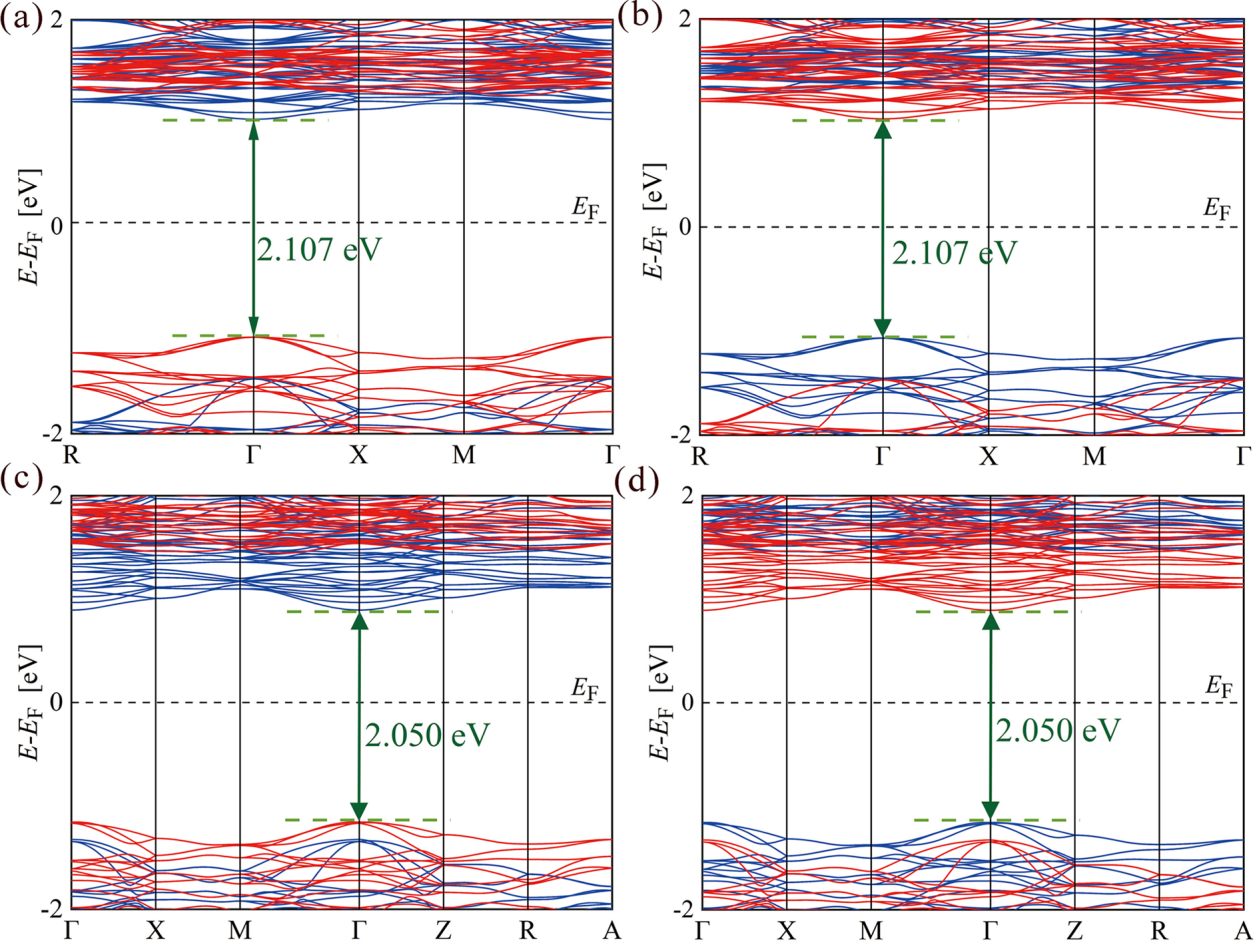
Electronic band structures of LFO along the high-symmetry paths: (*a*) |↑〉 on Fe1 and |↓〉 on Fe2 in the cubic model, (*b*) |↑〉 on Fe2 and |↓〉 on Fe1 in the cubic model, (*c*) |↑〉 on Fe1*a* and Fe1*b* and |↓〉 on Fe2 in the tetragonal model, and (*d*) |↑〉 on Fe2 and |↓〉 on Fe1*a* and Fe1*b* in the tetragonal model. The dashed lines at 0 eV indicate the Fermi energy. Spin-up and spin-down electrons are shown in red and blue, respectively.

The electronic band structures were calculated using the optimized structural and Hubbard parameters from self-consistent DFT calculations in both cubic and tetragonal models. The spin-up and spin-down states were calculated along the high-symmetry paths in Brillouin zones: *R*(0.5, 0.5, 0.5) → Γ(0, 0, 0) → *X*(0.5, 0, 0) → *M*(0.5, 0.5, 0) → Γ(0, 0, 0) in *P*4_3_32 and Γ(0, 0, 0) → *X*(0, 0.5, 0) → *M*(0.5, 0.5, 0) → Γ(0, 0, 0) → *Z*(0, 0, 0.5) → *R*(0, 0.5, 0.5) → *A*(0.5, 0.5, 0.5) in *P*4_3_2_1_2. The spin-up state in the octahedral sublattice (denoted the *B* sublattice) was set to |↑〉, while the spin-down state in the tetrahedral sublattice (denoted the *A* sublattice) was set to |↓〉, and then the electronic band gaps were calculated. The reverse configurations were also calculated (Fig. 6 ▸).

Regarding the *B* sublattice, their band structures for two reverse |↑〉 [Figs. 6 ▸(*a*) and 6 ▸(*c*)] and |↓〉 [Figs. 6 ▸(*b*) and 6 ▸(*d*)] states show direct band gaps. Our results do not agree with an indirect band-gap energy calculated for the spin-up state of Fe^3+^ in the octahedral *B* sublattice in a previous DFT study (Kim *et al.*, 2022 ▸). The DFT calculations performed in the present study clearly show direct band gaps for these two different ferrimagnetic spin configurations. Their identical magnitudes can be understood by the intrinsic symmetry of the spin-polarized Hamiltonian. These two magnetic states, which are the reverse of each other, belong to the same energetic and electronic solution for a collinear spin alignment in the DFT calculations, and are related by a global spin-flip symmetry. This results in a physically equivalent charge-density distribution. Therefore, in the subsequent calculations, we focused on the one case with |↑〉 in the *B* sublattice and |↓〉 in the *A* sublattice. The direct band-gap energy of 2.050 eV for the tetragonal model in *P*4_3_2_1_2 is slightly narrower than the value of 2.107 eV for the cubic model in *P*4_3_32. These band gaps calculated for the microstructure-free LFO crystalline structure are larger than the band-gap energy measured on nest-like crystallites of LFO (about 1.9 eV; Zhang & Zhang, 2016 ▸) and the direct band gap probed with LFO nanoparticles (1.6 eV; Chireh & Naseri, 2019 ▸; Li *et al.*, 2020 ▸).

**Figure 7:**
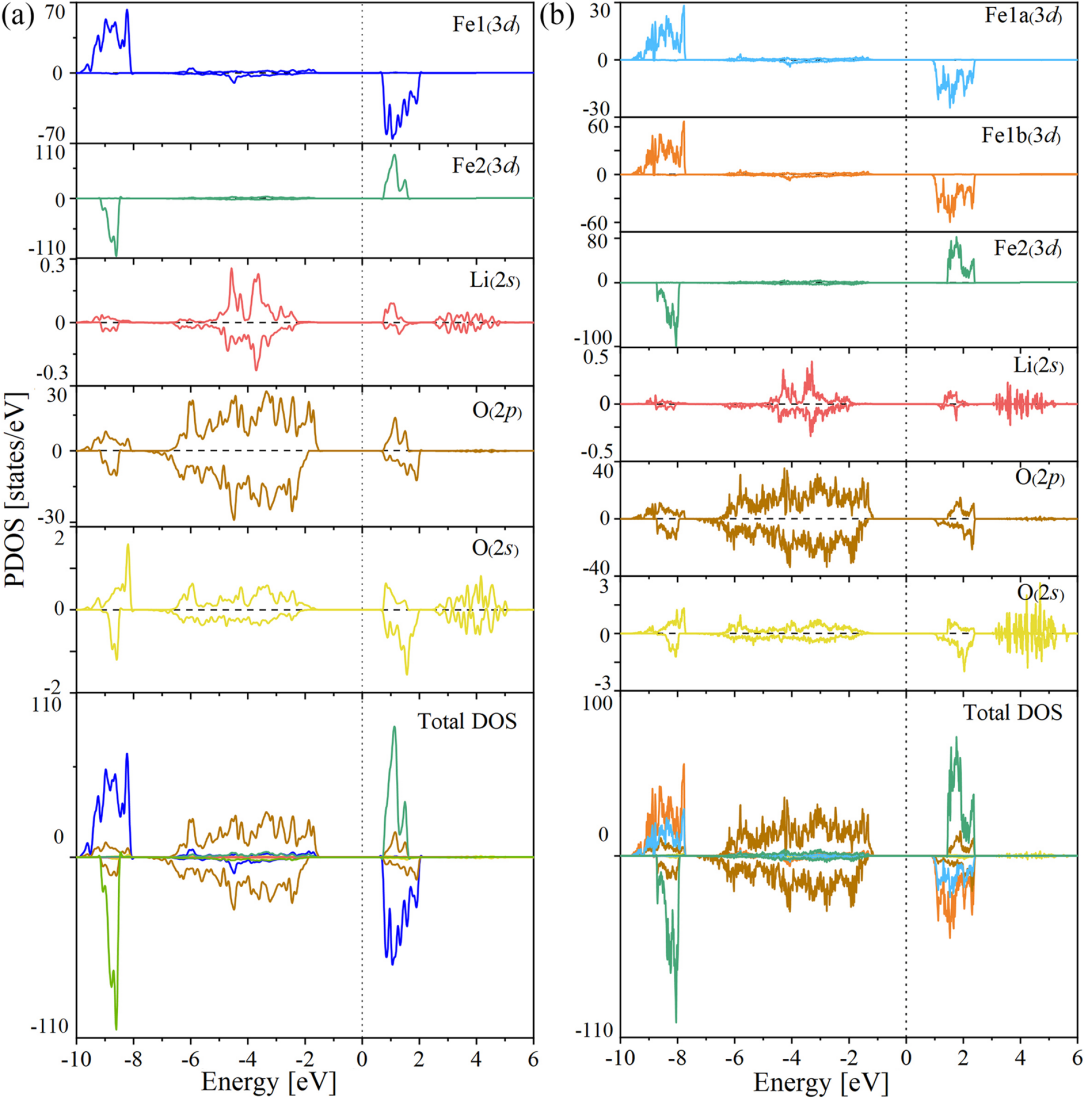
PDOS on all atomic sites of LFO, (*a*) in *P*4_3_32 and (*b*) in *P*4_3_2_1_2. The spin-up electron states are above 0 eV in the PDOS, while the spin-down electron states are below 0 eV. The Fermi energy levels are indicated with vertical dotted lines.

The spin-resolved projected density of states (PDOS) on each atomic site in the cubic and tetragonal models for LFO are graphically summarized in Fig. 7 ▸. The total DOS indicates the following:

(i) There is a strong hybridization between O 2*p* and Fe 3*d* orbitals in the valence bands. This hybridization is profound for the tetragonal model.

(ii) The conduction bands predominantly contribute to Fe 3*d* states. In particular, the conduction-band minimum is determined by the edge of the Fe 3*d* orbitals near the Fermi level. The spin-up electrons mainly originate from tetrahedral Fe sites, whereas the spin-down electrons are associated with octahedral Fe sites. A noticeable contribution of O 2*p* to these spin states is also seen in the total DOS.

(iii) The PDOS profiles on sites Fe1*a* and Fe1*b* in the tetragonal model differ from each other. This accords with the symmetry splitting with respect to the Fe1 site in the cubic model. The total DOS in the tetragonal model shows that the conduction-band minimum is predominantly contributed to by Fe 3*d* on the octahedral sites Fe1*a* and Fe1*b*. The minimum energy level of Fe 3*d* on Fe2 is much further from the Fermi level. This is highly possible due to a higher localization of electrons. In contrast, the Fe 3*d* orbitals on both Fe1 and Fe2 contribute to the conduction-band minimum in the cubic model. A higher localization of Fe 3*d* electrons in the tetragonal structure of LFO is associated with its *U* parameters being slightly larger than those in the cubic model (Table 1 ▸).

**Figure 8:**
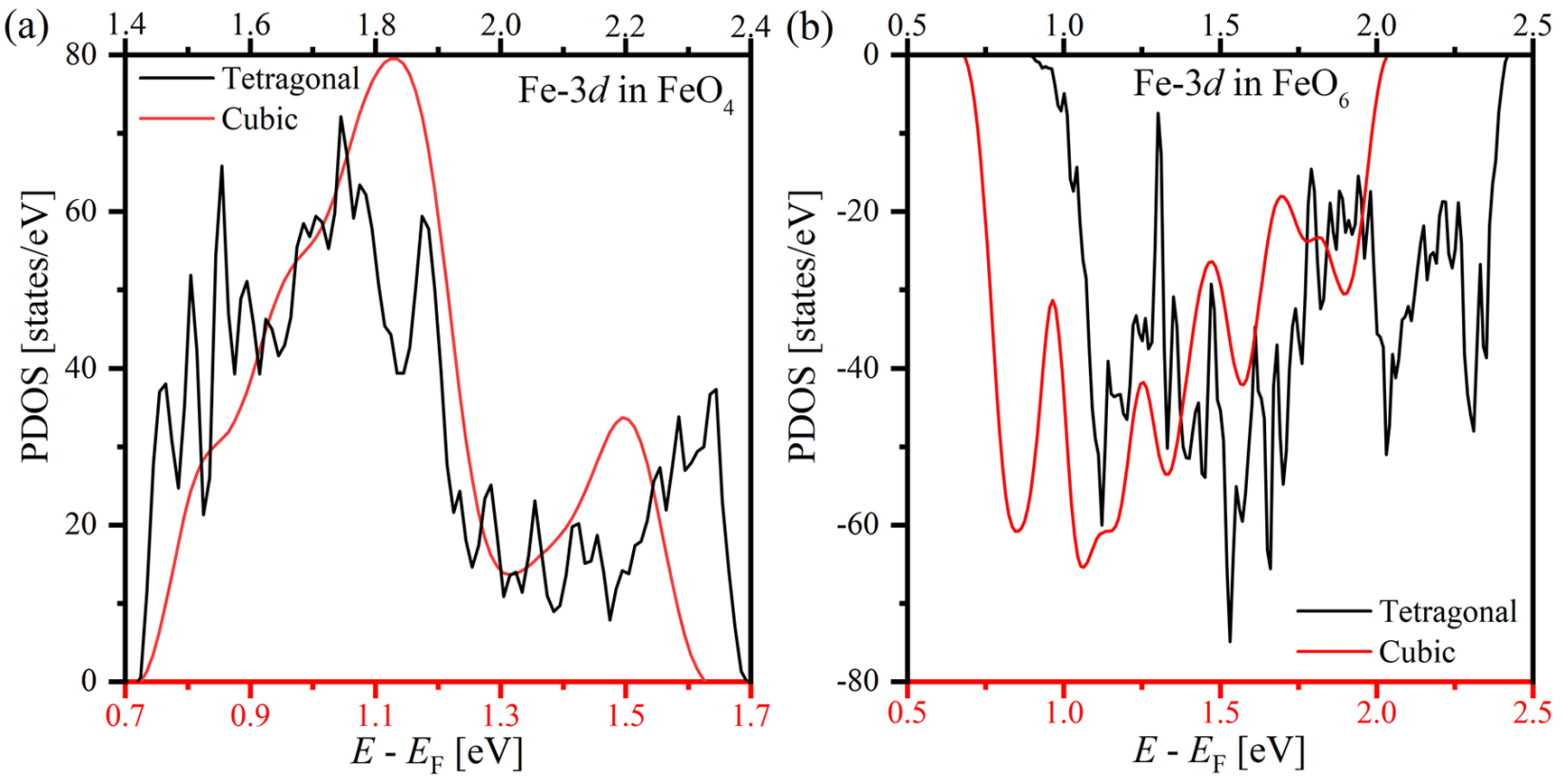
PDOS of the cubic model (red lines) against the *E* − *E*_F_ scale at the bottom, compared with PDOS of the tetragonal model (black lines) against the *E* − *E*_F_ scale at the top: (*a*) Fe 3*d* spin-up electrons on tetrahedral sites and (*b*) Fe 3*d* spin-down electrons on octahedral sites.

The PDOS profiles of Fe 3*d* on tetrahedral and octahedral sites are compared in Fig. 8 ▸, where the tetragonal model reveals a redistribution and extension of the unoccupied spin-up states, rather than a simple energy shift. Moreover, the PDOS profile of Fe 3*d* states on Fe2 exhibits a noticeably broader energy distribution in the tetragonal model compared with that in the cubic one. This suggests an increase in the bandwidth due to the electronic delocalization enhanced on the tetrahedral site Fe2 [Fig. 8 ▸(*a*)]. This contradicts the *U* values for a higher localization of Fe 3*d* electrons in the tetragonal model, as mentioned above. This conflict could be explained by *p*–*d* hybridization effects competing with on-site Coulomb interactions. The stability of LFO in the tetragonal structure might be enhanced by hybridization towards strong covalent interactions between Fe 3*d* electrons on Fe2 and O 2*p* electrons in the spin-up ferrimagnetic state.

### 4.3. Magnetic properties

The calculated cubic and tetragonal LFO models commonly show a total magnetization value of 2.5 μ_B_ per formula unit, consistent with experimentally determined values (Boyraz *et al.*, 2011 ▸; Kumawat *et al.*, 2021 ▸; Liu *et al.*, 2019 ▸). The calculated magnetic moments of Fe^3+^ on the octahedral site Fe1 are larger than those on the tetrahedral site Fe2 in the cubic model (Table 3 ▸). Similarly, the magnetic moments of Fe^3+^ on Fe1*a* (4.156 μ_B_) and Fe1*b* (4.149 μ_B_) are larger than that of the tetrahedral site Fe2 (4.044 μ_B_), corresponding to the high-spin state expected for LFO.

**Figure 9:**
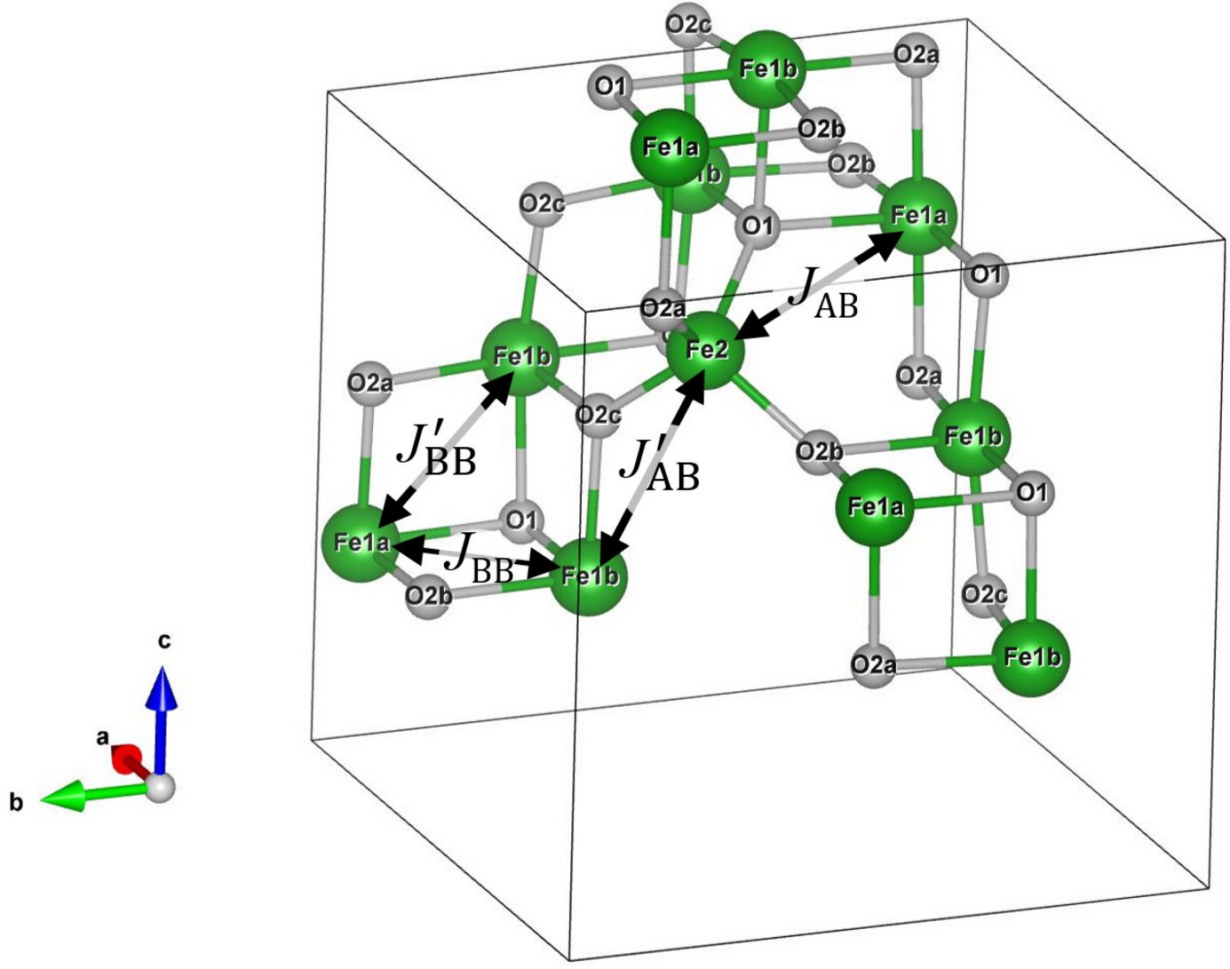
Nearest magnetic interaction paths of Fe–Fe within an interatomic distance of 3.4 Å in the calculated tetragonal model of LFO (see the main text).

**Figure 10:**
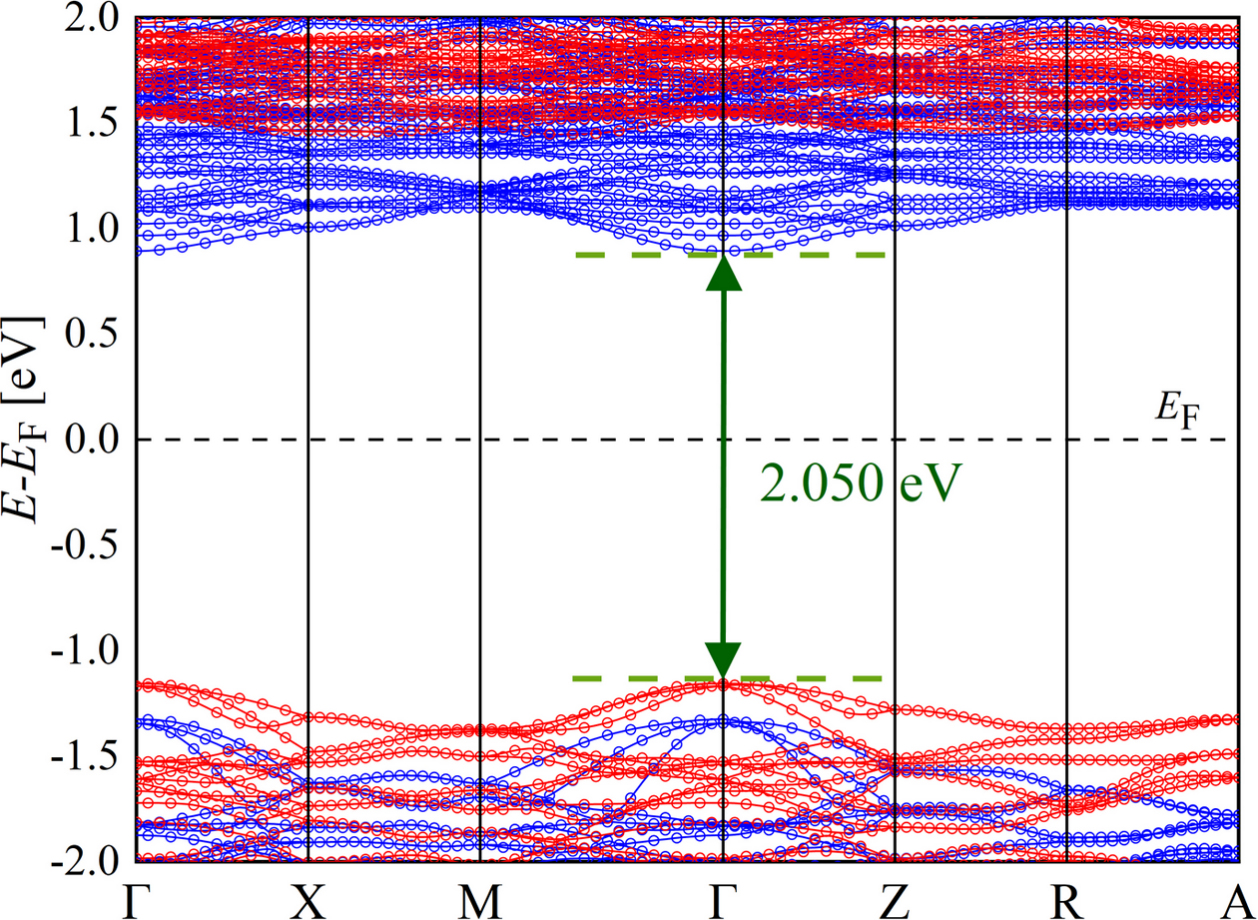
Electronic band structures calculated by Wannier functions (circles) for the tetragonal model of LFO compared with those in Bloch states (lines; see also Fig. 6). Spin-up and spin-down electrons are presented in red and blue, respectively.

We calculated the long-range magnetic interactions in LFO, resulting in negligibly small values near zero up to the fifth order. Hence, in this study, only the nearest magnetic interactions were considered. As shown in Fig. 9 ▸, there are four different nearest magnetic exchange interactions, *i.e.* the face-diagonal interaction *J*_*AB*_ between Fe (*A* sublattice) and Fe (*B* sublattice), the space-diagonal term *J*′_*AB*_, and *J*_*BB*_ and *J*′_*BB*_ which are the corresponding face- and space-diagonal terms within the *B* sublattice. Their magnitude ratios affect the degree and fashion of tetragonality in spinels (Menyuk *et al.*, 1962 ▸). Therefore, to clarify the origin of the tetragonal lattice distortion *a*/*c* > 1 feasible in the ground state of LFO (Table 1 ▸), these magnetic exchange interaction parameters were calculated using SSSP pseudopotentials plus PBEsol. The resulting magnetic exchange parameters are highly reliable, as the band structure calculated using Wannier functions matches well that using Bloch states (Fig. 10 ▸).

To construct the effective spin Hamiltonian, magnetic exchange interactions were first evaluated using the magnetic moments projected onto Fe alone. In this case, the resulting exchange parameters correspond to effective Fe–Fe inter­actions, where O is not treated as an independent magnetic degree of freedom; the magnetic interaction contribution of O, arising from Fe–O–Fe superexchange processes, is implicitly included in the effective Fe–Fe exchange parameters. The total Hamiltonian (*H*_total_) is expressed by the static Hamilton­ian *H*_static_ with the effective interaction *J*_eff_, 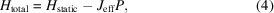
where *P* is the spin exchange operator. This formalism allows us to derive a minimal Heisenberg model for the Fe sub­lattices. In another case, to prove the hybridization, particularly in Fe_2_O_4_ as mentioned in Section 4.2, the magnetic moments were projected onto both Fe and O, with the spin polarization of ligand oxygens explicitly taken into account. As a result, a part of the magnetic coupling, which was incorporated into the effective Fe–Fe interaction in the previous case, is redistributed into Fe–O–Fe superexchange terms. The contribution of Fe–O–Fe superexchange inter­actions to *J*_eff_ corresponds to *p*–*d* hybridization. The magnetic moments for both projection cases are listed in Table 4 ▸, along with the four nearest magnetic interactions calculated using Wannier functions. The magnitude differences on the octahedral sites Fe1*a* and Fe1*b* between the two projection cases are similar to each other. In contrast, the magnetic moment on Fe2 projected onto Fe (−4.9928 μ_B_) is much larger than that from the projection onto both Fe and O (−4.1476 μ_B_). Furthermore, the latter is very similar to the magnetic moment magnitude on Fe2 calculated in Bloch states (−4.044 μ_B_). This large discrepancy (Δ) of 0.8542 μ_B_ on Fe2 between the two projection cases points to strong Fe–O–Fe superexchange interactions in Fe2O_4_. The superexchange effect in Fe1*a*O_6_ and Fe1*b*O_6_ might be smaller, as indicated by the respective low Δ values of 0.0845 μ_B_ and 0.1689 μ_B_.

The suggested strong Fe–O–Fe superexchange interactions are consistent with the pronounced *p*–*d* hybridization observed in the PDOS of the Fe 3*d* and O 2*p* states (Fig. 7 ▸). The Goodenough–Kanamori–Anderson (GKA) rules (Anderson, 1959 ▸; Kanamori, 1957 ▸; Kanamori, 1959 ▸) state that the sign and strength of superexchange interactions are influenced by ∠(Fe–O–Fe) angles and the orbital occupancy. The superexchanges Fe1*a*(↑)–O(↑)–Fe1*b*(↑) and Fe1*b*(↑)–O(↑)–Fe1*b*(↑) occur over ∠(Fe–O–Fe) ≃ 90° (Table A.III). According to the GKA rules, the bonding geometry of ∠(Fe–O–Fe) ≃ 90° favours ferromagnetic interactions due to the orthogonality of the involved orbitals. In contrast, the Fe1*b*(↑)–O(↑)–Fe2(↓) and Fe1*a*(↑)–O(↑)–Fe2(↓) pathways exhibit ∠(Fe–O–Fe) ≫ 90°. This favours antiferromagnetic coupling due to maximal overlap of the half-filled Fe 3*d* orbitals with the O 2*p* orbitals. The inclusion of O 2*p* states accounts for the strong Fe 3*d*–O 2*p*–Fe 3*d* hybridization on Fe2O_4_, where superexchange interactions are significant.

When applying the Yafet–Kittel model for spinels, the magnetic energy for a tetragonal distortion can be characterized by three dimensionless parameters, which describe the relative exchange interaction strength and the magnetic anisotropy (Menyuk *et al.*, 1962 ▸). These parameters are defined as 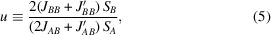

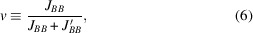

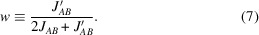
The spin vectors *S*_*B*_ = 5/2 and *S*_*A*_ = −5/2 correspond to the respective high-spin states of Fe^3+^ in the *B* and *A* sublattices. Using the calculated parameters (*J*_*BB*_ = 5.3791 meV, *J*′_*BB*_ = 5.1697 meV, *J*_*AB*_ = −1.4478 meV and *J*′_*AB*_ = −1.8574 meV), the magnitudes of *u*, *v* and *w* were calculated to determine the tetragonality of LFO as follows: 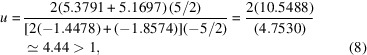

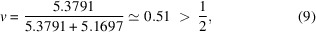

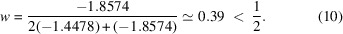
The parameter *u* > 1 characterizes the relative strength of ferromagnetic interactions in the *B* sublattice in comparison with the antiferromagnetic interactions between the *A* and *B* sublattices. The condition *v* > 1/2 indicates that ferromagnetic interactions within the *B* sublattice are stronger on the *ab* plane than those parallel to the *c* direction; *w* < 1/2 implies that antiferromagnetic interactions between the *A* and *B* sub­lattices along the *c* axis are stronger than those in the *ab* plane. These parameters explain how a total energy lowering is realized by the tetragonal distortion *a* > *c* in LFO, induced by decreasing ferromagnetic interactions and increasing antiferromagnetic interactions along *c*, simultaneously. This explains the feasibility of the tetragonal structure model for LFO with an *a*/*c* ratio slightly larger than 1 (Table 1 ▸). In summary, the tiny tetragonality of LFO has a magnetic origin from strong ferromagnetic interactions in the *B* sublattice competing with the anisotropy of antiferromagnetic inter­actions, including the Fe–O–Fe superexchange.

## 5. Conclusions

There has been a need to find the true structure of α-LiFe_5_O_8_ (denoted LFO) compatible with a collinear ferrimagnetic spin arrangement on the crystallographic *c* axis. Thus, the aim of this study was to determine the true ground-state symmetry of LFO to re-examine and re-interpret its structural and physical properties. In a comprehensive first-principles investigation on the atomic, electronic and magnetic structures of LFO, we have dealt with two structural models in the cubic space group *P*4_3_32, which has been known to be true for LFO to date, and in its tetragonal subgroup *P*4_3_2_1_2. Results from SSSP+PBEsol+*U* calculations suggest that the tetragonal lattice is more favourable than the ideal cubic one within the calculation accuracy.

Our magnetic structure simulations show that LFO inherits the ferrimagnetic structure in the tetragonal magnetic space group *P*4_3_
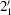
2′, which is consistent with the refined model from our recent analysis with HRNPD (to be published). This ferrimagnetic structure model comprises a spin-down tetrahedral diamond lattice (*A* sublattice) and a spin-up octahedral hyper-Kagome lattice (*B* sublattice). The calculated nearest-neighbour effective magnetic exchange interactions indicate that ferromagnetic interactions dominate over antiferro­mag­netic interactions in this ferrimagnetic arrangement. The new and insightful findings in this relevant prototype for MF-PVs are highlighted as follows:

(i) there is a tiny tetragonal lattice distortion *a*/*c* > 1 with *a* = 8.3528 (2) Å and *c* = 8.3511 (1) Å;

(ii) this faint tetragonality has a magnetic origin;

(iii) the Fe–O–Fe magnetic superexchange interaction is consistent with Fe 3*d*–O 2*p* hybridization, particularly strong in Fe2O_4_ tetrahedra;

(iv) the direct band gap of 2.050 eV for microstructure-free LFO in the tetragonal model is smaller than that in the cubic model.

As a relevant aspect, the extremely small tetragonality of LFO (and its variants) might be considered for developing LFO-based MF-PVs. Slight shifts in atomic positions caused by magnetic interactions in (multi)ferroic structures could be verified with greater accuracy at sub-picometre resolution using new resonant X-ray diffraction techniques (Richter *et al.*, 2018 ▸), *e.g.* YMn_2_O_5_ (Weigel *et al.*, 2024 ▸). On the other hand, to realize LFO-type PVs in industrial applications, further experimental and theoretical work is necessary to determine their dynamic magnetic charges (Ye & Vanderbilt, 2014 ▸) and the shift-current response under external fields (Jalaja & Dutta, 2015 ▸; Gong *et al.*, 2024 ▸; Burger *et al.*, 2019 ▸).

Last but not least, the present study could serve as a basis for enhancing PV effectiveness for LFO-type MFs. For instance, Al-doped LFO solid-solution compounds that exhibit a relatively strong tetragonal distortion (Inckemann *et al.*, 2025 ▸) show significantly lower band-gap energies. Details will be reported elsewhere.

## Supplementary Material

Supplementary File. DOI: 10.1107/S1600576726006709/po5178sup1.pdf

## Figures and Tables

**Table 1:** Results from DFT calculations for the ground state of LFO in both cubic and tetragonal models by the SSSP+PBEsol method

	Crystal system							
	Cubic				Tetragonal			
Space group	*P*4_3_32				*P*4_3_2_1_2			
Unit-cell parameters (Å)	*a* = 8.3550 (1)				*a* = 8.3528 (2), *c* = 8.3511 (1)			
*V*_0_ (Å^3^)	583.2293 (3)				582.6501 (3)			

	Site	Coordinates			Site	Coordinates		
	(Wyckoff)	*x*	*y*	*z*	(Wyckoff)	*x*	*y*	*z*
	Fe1 (12*d*)	0.1250	0.3644 (1)	0.8856 (1)	Fe1*a* (4*a*)	0.6173 (3)	0.6173 (3)	0
					Fe1*b* (8*b*)	0.8750 (1)	0.3674 (1)	0.0076 (2)
	Fe2 (8*c*)	0.9947 (1)	0.9947 (1)	0.9947 (1)	Fe2 (8*b*)	0.2523 (1)	0.0023 (1)	0.6227 (2)
	Li (4*b*)	0.8750	0.3750	0.1250	Li (4*a*)	0.1250	0.1250	0
	O1 (8*c*)	0.3897 (1)	0.3897 (1)	0.3897 (1)	O1 (8*b*)	0.1349 (1)	0.3849 (1)	0.5099 (1)
	O2 (24*e*)	0.1212 (1)	0.1261 (1)	0.3817 (1)	O2*a* (8*b*)	0.8682 (1)	0.1269 (1)	0.5084 (1)
					O2*b* (8*b*)	0.1334 (1)	0.1183 (1)	0.2519 (1)
					O2*c* (8*b*)	0.8769 (1)	0.3835 (1)	0.2433 (1)

*U* (eV)	Fe1	5.0561			Fe1*a*	5.0724		
					Fe1*b*	5.0839		
	Fe2	5.1556			Fe2	5.1668		
Total energy (Ry)	−7911.0052				−7911.0057			
*B*_0_ (GPa)	168.30 ± 0.04				166.20 ± 0.06			
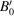 (d*B*_0_/d*P*)	4.256 ± 0.005				4.221 ± 0.003			

**Table 2:** Average polyhedral degrees of distortion in two different theoretical models for LFO

					Electric dipole moment (e Å)			
Model	Polyhedron	Site	Volume (Å^3^)	Distortion (%)	*x*	*y*	*z*	Modulus (e Å)
Cubic	LiO_6_	Li1	12.434 (1)	0.2 (1)	0	0	0	0
	FeO_6_	Fe1	10.873 (1)	0.5 (1)	−0.8572 (1)	0	0.8572 (1)	1.21 (1)
	FeO_4_	Fe2	3.527 (1)	0.3 (1)	−0.0157 (1)	0.0157 (1)	−0.0157 (1)	0.03 (1)

Tetragonal	LiO_6_	Li1	12.415 (1)	0.4 (1)	1.1409 (1)	1.1409 (1)	0	1.61 (1)
	FeO_6_	Fe1*a*	11.355 (1)	1.8 (1)	−0.2121 (1)	−0.2121 (1)	0	0.30 (1)
	FeO_6_	Fe1*b*	11.188 (1)	3.3 (1)	−1.1360 (1)	0.6582 (1)	0.6013 (1)	1.44 (1)
	FeO_4_	Fe2	3.344 (1)	0.9 (1)	0.0100 (1)	−0.4243 (1)	0.1303 (1)	0.44 (1)

**Table 3:** Magnetic moments of Fe^3+^ along the *c* axis in the LFO structure models in *P*4_3_32 and *P*4_3_2_1_2, based on Bloch states

Space group	Atomic site	Magnetic moment (μ_B_)
*P*4_3_32	Fe1	4.156
	Fe2	−4.065

*P*4_3_2_1_2	Fe1*a*	4.156
	Fe1*b*	4.149
	Fe2	−4.044

**Table 4:** Effective magnetic exchange interaction parameters (meV) and magnetic moments (μ_B_) in the tetragonal LFO model, calculated using Wannier functions

Effective magnetic exchange interaction		Atomic site	Magnetic moment projected on Fe	Magnetic moment projected on Fe and O
*J*_*BB*_	5.3791	Fe1*a*	4.3300	4.2455
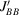	5.1697	Fe1*b*	4.4070	4.2381
*J*_*AB*_	−1.4478	Fe2	−4.9928	−4.1476
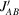	−1.8574	O1	–	0.1344
		O2*a*	–	0.0642
		O2*b*	–	0.0519
		O2*c*	–	0.0363
