# Supplementary material for: Tetragonal distortion of the multiferroic compound α-LiFe5O8 from first-principles calculations
Source: J Appl Crystallogr. 2026 Jul 31;59(Pt 4):1307–18. doi: 10.1107/S1600576726006709 (PMC13440051; doi:10.1107/S1600576726006709)
Supplement: Supplementary file 1 [file j-59-01307-sup1.pdf]

JOURNAL OF
APPLIED
CRYSTALLOGRAPHY

**Volume 59 (2026)**

**Supporting information for article:**

# Tetragonal distortion of the multiferroic compound α-$LiFe_5O_8$ from first-principles calculations

**Yaoshi Cheng and So-Hyun Park**

TABLE A.I. Results from DFT calculations for the ground state of LFO in the cubic and tetragonal models from the SSSP+PBE method.

| Crystal system | cubic | | | | tetragonal | | | |
|---|---|---|---|---|---|---|---|---|
| Space group | $\boldsymbol{P}4_332$ | | | | $\boldsymbol{P}4_32_12$ | | | |
| Lattice parameters [Å] | a = 8.4553(3) | | | | a = 8.4541(2)<br>c = 8.4531(1) | | | |
| $V_0$ [Å$^3$] | 604.4871(3) | | | | 604.1583(3) | | | |
| | Site (Wyckoff) | Coordinates | | | Site (Wyckoff) | Coordinates | | |
| | | x | y | z | | x | y | z |
| | Fe1 (12d) | 0.1250 | 0.3644(1) | 0.8856(1) | Fe1a (4a) | 0.6174(1) | 0.6174(1) | 0 |
| | | | | | Fe1b (8b) | 0.8750(1) | 0.3675(1) | 0.0075(1) |
| | Fe2 (8c) | 0.9947(1) | 0.9947(1) | 0.9947(1) | Fe2 (8b) | 0.2522(1) | 0.0022(1) | 0.6227(1) |
| | Li (4b) | 0.8750 | 0.3750 | 0.1250 | Li (4a) | 0.1250 | 0.1250 | 0 |
| | O1 (8c) | 0.3897(1) | 0.3897(1) | 0.3897(1) | O1 (8b) | 0.1349(1) | 0.3849(1) | 0.5098(1) |
| | O2 (24e) | 0.1212(1) | 0.1261(1) | 0.3817(1) | O2a (8b) | 0.8679(1) | 0.1274(1) | 0.5082(1) |
| | | | | | O2b (8b) | 0.1332(1) | 0.1179(1) | 0.2523(1) |
| | | | | | O2c (8b) | 0.8774(1) | 0.3833(1) | 0.2430(1) |
| $U$ [eV] | Fe1 5.0308<br>Fe2 5.1342 | | | | Fe1a 5.0619<br>Fe1b 5.0754<br>Fe2 5.1494 | | | |
| Total energy [Ry] | -7974.696 | | | | -7974.738 | | | |
| $B_0$ [GPa] | $169.40 \pm 0.06$ | | | | $168.50 \pm 0.08$ | | | |
| $B_0'$ (d$B_0$/dP) | $4.273 \pm 0.007$ | | | | $4.212 \pm 0.003$ | | | |

TABLE A.II. Interatomic distances and angles in the cubic model for LFO (SG: $\boldsymbol{P}4_332$)

| Site | Type | Ligand | Interatomic distance [Å] | Bonds | Interatomic angle [°] |
|---|---|---|---|---|---|
| Li | Li | O2 (×6) | 2.099(1) | O2-Li-O2 | 87.24(1) |
| | | | | O2-Li-O2 | 91.80(1) |
| | | | | O2-Li-O2 | 93.74(1) |
| Fe1 | Fe | O1 (×2) | 2.0589(1) | O1-Fe1-O1 | 92.08(1) |
| | | O2 (×2) | 2.0264(1) | O2-Fe1-O2 | 92.92(1) |
| | | O2 (×2) | 1.9974(1) | O2-Fe1-O2 | 99.12(1) |
| | | | | O2-Fe1-O2 | 92.75(1) |
| | | | | O2-Fe1-O1 | 90.69(1) |
| | | | | O2-Fe1-O1 | 83.23(1) |
| | | | | O2-Fe1-O1 | 84.52(1) |
| Fe2 | Fe | O1 (×1) | 1.9450(1) | O1-Fe2-O2 | 110.24(1) |
| | | O2 (×3) | 1.8872(1) | O2-Fe2-O2 | 108.70(1) |
| | | | | Fe1-O2-Fe1 | 97.96(1) |
| | | | | Fe1-O1-Fe1 | 94.29(1) |
| | | | | Fe1-O2-Fe1 | 97.96(1) |
| | | | | Fe1-O1-Fe2 | 122.17(4) |
| | | | | Fe1-O2-Fe2 | 123.32(6) |

TABLE A.III. Interatomic distances and angles of the tetragonal model for LFO (SG: $\boldsymbol{P}4_32_12$)

| Site | Type | Ligand | Interatomic distance [Å] | Bonds | Interatomic angle [°] |
|---|---|---|---|---|---|
| Li | Li | O2a (×2) | 2.1106(1) | O2a-Li-O2a | 93.26(1) |
| | | O2b (×2) | 2.1438(1) | O2a-Li-O2b | 93.78(1) |
| | | O2c (×2) | 2.1097(1) | O2a-Li-O2c | 86.48(1) |
| | | | | O2b-Li-O2c | 93.25(1) |
| | | | | O2c-Li-O2c | 93.79(1) |
| | | | | O2b-Li-O2c | 86.48(1) |
| | | | | O2b-Li-O2a | 86.48(1) |
| Fe1a | Fe | O1 (×2) | 2.0750(1) | O1-Fe1a-O1 | 91.18(1) |
| | | O2a (×2) | 2.0581(1) | O1-Fe1a-O2a | 89.97(1) |
| | | O2b (×2) | 2.0011(1) | O1-Fe1a-O2a | 84.29(1f) |
| | | | | O1-Fe1a-O2b | 85.64(1) |
| | | | | O2b-Fe1a-O2b | 97.69(1) |
| | | | | O2a-Fe1a-O2b | 92.72(1) |
| | | | | O2a-Fe1a-O2b | 92.68(1) |
| Fe1b | Fe | O1 | 2.0736(1) | O1-Fe1b-O1 | 91.18(1) |
| | | O1 | 2.0629(1) | O1-Fe1b-O2a | 85.64(1) |
| | | O2a | 2.0098(1) | O1-Fe1b-O2b | 89.99(1) |
| | | O2b | 2.0476(1) | O1-Fe1b-O2b | 84.29(1) |
| | | O2c | 2.0315(1) | O1-Fe1b-O2c | 89.98(1) |
| | | O2c | 2.0073(1) | O1-Fe1b-O2c | 84.28(1) |
| | | | | O1-Fe1b-O2c | 85.64(1) |
| | | | | O2a-Fe1b-O2b | 92.71(1) |
| | | | | O2a-Fe1b-O2c | 92.68(1) |
| | | | | O2a-Fe1b-O2c | 97.68(1) |
| | | | | O2b-Fe1b-O2c | 92.67(1) |
| | | | | O2c-Fe1b-O2c | 92.71(1) |
| Fe2 | Fe | O1 | 1.9197(1) | O1-Fe2-O2a | 109.18(1) |
| | | O2a | 1.8737(1) | O1-Fe2-O2b | 109.16(1) |
| | | O2b | 1.8601(1) | O1-Fe2-O2c | 109.17(1) |
| | | O2c | 1.8332(1) | O2a-Fe2-O2b | 109.77(1) |
| | | | | O2a-Fe2-O2c | 109.77(1) |
| | | | | O2b-Fe2-O2c | 109.78(1) |
| | | | | Fe1a-O1-Fe1b | 91.42(1) |
| | | | | Fe1a-O2a-Fe1b | 93.77(1) |
| | | | | Fe1a-O2b-Fe1b | 95.38(1) |
| | | | | Fe1a-O1-Fe2 | 122.99(1) |
| | | | | Fe1a-O2a-Fe2 | 121.79(1) |
| | | | | Fe1a-Ob-Fe2 | 124.95(1) |
| | | | | Fe1b-O2a-Fe2 | 119.89(1) |
| | | | | Fe1b-O2b-Fe2 | 123.87(1) |
| | | | | Fe1b-O2c-Fe2 | 126.33(1) |
